# Leisure Possibilities of Adults Experiencing Poverty: A Community-Based Participatory Study

**DOI:** 10.1177/00084174211073266

**Published:** 2022-02-14

**Authors:** Pamela Cantor, Monika Polakowska, Amanda Proietti, Victor Tran, Jonathan Lebire, Laurence Roy

**Keywords:** Occupational engagement, Participatory research, Poverty, Social determinants of health, Déterminants sociaux de la santé, engagement occupationnel, pauvreté, recherche participative

## Abstract

**Background.** Poverty disproportionally affects persons with disabilities, elderly individuals and racialized groups. Leisure, play and rest are not prioritized in either services for or research with people living in poverty. **Purpose.** This study aims to examine the facilitators and barriers to participation in meaningful leisure activities for adults living in poverty. **Method.** We used community-based participatory research and art-based elicitation strategies with 39 service users at a food security organization. **Findings.** Individuals experiencing poverty value and engage in a variety of free and affordable leisure activities, but they are not afforded the necessary leisure opportunities, accommodations and supports as the general population. We co-created a map of local leisure resources to foster collective capacity in leisure planning, and to support organizations working with this population. **Implications.** Occupational therapists can work alongside members of underserved communities to uncover and address the systemic and local contextual barriers to engagement in leisure activities.

## Résumé

**Description.** La pauvreté touche de manière disproportionnée les personnes en situation de handicap, les personnes âgées et les groupes racisés. Les loisirs, le jeu et le repos ne sont jamais prioritaires parmi les services offerts aux personnes en situation de pauvreté ou les recherches qui les concernent. **But.** La présente étude vise à examiner les facilitateurs et les obstacles à la participation à des activités de loisir significatives pour les adultes qui vivent dans la pauvreté. **Méthodologie.** Nous avons réalisé une recherche participative en milieu communautaire et utilisé des stratégies de stimulation de la conversation basées sur les arts auprès de 39 utilisateurs et utilisatrices des services d’un organisme de sécurité alimentaire. **Résultats.** Les personnes en situation de pauvreté participent à diverses activités de loisir gratuites ou abordables et y accordent de l’importance, mais elles n’ont pas accès aux mêmes possibilités, aux mêmes accommodements et au même soutien que la population en général. Nous avons cocréé une carte des ressources locales en matière de loisir afin de stimuler la capacité collective à planifier des loisirs, et de soutenir les organismes qui travaillent auprès de cette population. **Conséquences.** Les ergothérapeutes peuvent travailler auprès de membres de communautés mal desservies de manière à faire ressortir les obstacles systémiques et contextuels à la participation aux activités de loisir et y trouver des solutions.

## Introduction

Poverty is often understood as economic deprivation where the lack of income is deemed to be the most characteristic feature in defining the term (Employment and Social Development Canada, 2016). In 2017, 9.5% of the Canadian population was living below Canada’s Official Poverty Line (Statistics Canada, 2019). Nonetheless, poverty is a multidimensional concept that encompasses a myriad of social, cultural and political aspects extending beyond the simple lack of money resources (Canadian Poverty Institute, n.d.), including material, social and spiritual deprivation. As such, the number of Canadians experiencing poverty may be much greater. While a small reduction in the number of Canadians living below poverty line has been observed in the 2016–2019 years (Statistics Canada, 2019), poverty is expected to rise in the aftermath of the COVID-19 pandemic (Lemieux et al., 2020).

Not only is poverty a multidimensional concept and multi-faceted experience in itself, its causes and roots are also complex and multi-factorial. In Canada specifically, the move to neoliberal policies and practices, particularly in the management of welfare, social and health services, has contributed to a rise in poverty and unemployment (Smith-Carrier, 2017). The interplay of structural factors (such as the nature and availability of employment, inflation rates, housing market conditions, immigration policies and availability of needed services and supports) and personal situations (such as single parenthood, injury, poor health or disability, immigration, trauma or violence) contribute to creating and maintaining poverty (Employment and Social Development Canada, 2016; Smith-Carrier, 2017). As such, women, people with disabilities, single parents, Indigenous peoples, immigrants and older adults are more susceptible to live in poverty (Canada Without Poverty, 2019; Employmentand Social Development Canada, 2016; Smith-Carrier, 2017), and are disproportionally affected by the COVID-19 crisis (Beland et al., 2020).

The effects of poverty manifest themselves in individuals’ lives through food and housing insecurity, and markedly poorer health than the general population, such that a 21-year difference in life expectancy can be observed between the wealthiest and the poorest in certain regions of Canada (Buist, 2015). The impact of poverty on the health and wellbeing of individuals has been extensively acknowledged (Gessler et al., 2011; Silva et al., 2016; Shahidi et al., 2019; McCartney et al., 2019). Given the needs of this group and the considerable barriers to health and social care they face (Gessler et al., 2011), many individuals living in poverty thus rely on the community sector to fulfil their needs.

Beyond its health effects, poverty is known to affect the occupations of individuals and groups (Bazyk & Bazyk, 2009; Galvaan, 2012; Hammell, 2015). Occupational justice alludes to the right to engage in meaningful and purposeful occupations. As such, an occupational justice perspective recognizes individuals as occupational beings that have unique occupational needs and require opportunities to engage in meaningful activities due to their diverse personal and situational circumstances and capacities (Townsend & Wilcock, 2004; Wilcock, 2006; Wilcock & Townsend, 2009). Occupational therapists can play a crucial role in addressing poverty-based occupational injustices (Chapleau et al., 2012; Illman et al., 2013), yet much of their documented work with individuals in situations of poverty is centred on skills and vocational training (Peter & Polgar, 2020; Roy et al., 2017). This focus is in concordance with the importance attributed to productive work and neoliberal values in western society (Bundy, 1993; Canadian Association of Occupational Therapists, 1997; Ingham, 1986; Samdahl, 1991) and in the OT profession (Gerlach et al., 2018; Farias & Rudman, 2019), to the detriment of activities categorized as self-care or leisure (Peter & Polgar, 2020).

In a similar fashion, much of the scholarly occupational therapy and science work in the field of poverty has focused on health and health equity (see for instance Gerlach, 2015) rather than on the occupational experiences of community members, and very limited empirical research has been conducted (Heatwole Shank et al., 2019). More research has been conducted on the intersection of leisure and poverty in the field of leisure sciences. From a biomedical or psychological perspective, leisure engagement has shown to improve health, wellbeing and life satisfaction (Kim et al., 2015), and to foster a positive sense of identity, confidence and self-efficacy (Haggard & Williams, 1992). From a critical perspective, leisure scholars have argued that leisure, and particularly recreational programs, have been used to regulate segments of the population perceived as “problematic,” including those in situations of poverty (Tink et al., 2019). Conversely, engagement in some forms of leisure, particularly community or collective ones, has been described as a means for democracy and expression of citizenship (Hemingway, 1999), and has been linked to increased social capital for underserved communities (Glover, 2004). However, the literature in both leisure and occupational science has been mostly theoretical, and empirical research is lacking to document how adults experiencing poverty engage in leisure, what barriers and facilitators to participation in meaningful leisure activities exist, and how service providers do and could promote participation in leisure activities while considering the specific needs, desires and rights of people experiencing poverty.

### The Current Study

The study described in this paper was initiated by the first author, who was at the time a Masters’ student in occupational therapy at a university in a large Canadian urban centre, and connected with an outreach worker in a food security community organization in the same city. The pre-existing relationship between these two individuals led to the development of a community-based participatory study co-led by a group of four Masters students, their supervisor (a faculty member with expertise in community health), and the community organization as a whole, including a coordinator, frontline workers, and service users, during May to July of 2019. Various members of the community organization had previously identified needs related to engagement in leisure and recreation among service users; however, lack of funding and knowledge of leisure resources among staff members impeded attempts to address this issue.

The aims of the study, established in a collaborative way between the research team and community organization members, were thus to describe the current leisure occupation profiles of adults experiencing poverty and to identify the barriers and facilitators to participation in meaningful leisure activities. Another objective of the study was to co-create a resource designed to facilitate engagement in leisure occupations for this group, and to foster self-efficacy and reflexivity in leisure planning.

## Methods

### Research Paradigm

The research paradigm guiding our process is critical theory, and we particularly mobilize the work of Nancy Fraser and others, in our understanding of the social processes that lead to injustice, both social and occupational (Fraser, 1996; Fraser & Honneth, 2003). In her work, Fraser attempts to reconcile two explanatory models of injustice – namely, injustice as a problem of distribution and injustice as a problem of recognition – and argues that we need to recognize the differentiated yet interpenetrating ways in which the two create situations of injustice. For instance, from Fraser’s perspective, women’s poverty is both a problem of economic distribution, linked to the fundamental division between paid “productive” labour and unpaid domestic labour, but also a problem of gender norms and status subordination. Although Fraser’s work has been particularly linked to feminist theories, it has been used to understand various types of injustices and sources of oppression, including in the field of disability (Danermark & Gellerstedt, 2004). Here, critical theory serves to question assumptions around who has the right to access leisure, as well as the social, cultural and economic attitudes that create barriers to leisure for adults living in poverty (Hoffman, 1987; Salkind, 2010).

### Ethical Considerations

The research ethics board of McGill University approved this study. Written informed consent was obtained from all participants. In this study, we link ethical considerations to the chosen methodology of community-based participatory research (CBPR; Wallerstein & Duran, 2010), particularly its feature as an engaged and reflexive practice. Engagement in this study manifested itself through the long-term presence and participation of the student researchers in the community organization’s space and activities. The student researchers attended the community organization 2 to 3 days per week between May and July 2019. During that time, student researchers dined with members of the organizations to build rapport, and to clarify the purpose of their presence at the organization. They also met regularly with their internal contact at the community organization (who is a co-author on this paper) for discussions, training and information. The student researchers continued to visit the organizations on a weekly basis once data collection was complete, as a form of member checking and to preserve connections with members. Engagement also manifested itself through the choice of arts-based elicitation strategies, as community members (staff and service users) expressed the importance to not only discuss leisure exclusion as a form of injustice, but to actually create transformative opportunities for leisure engagement through the research process.

Reflexive practice occurred through regular individual and collective reflections and dialogue on positionality, power dynamics and assumptions with regards to the phenomenon of poverty. The research team engaged in journaling through memos, as well as regular reflexive meetings, two to three times per week. Student and faculty researchers came from a mix of socio-economic and cultural backgrounds; notably, three out of five individuals in the research team identified as coming from a working-class background or carrying experiential knowledge of poverty. One element that emerged from reflecting on our individual and group positionality (for instance, as situated, at the time of the study, within a historically privileged academic institution) was the need to consider poverty from an intersectional perspective (Collins & Bilge, 2016; Crenshaw, 1989). Experiences of economic deprivation within the research team often intersected with social identities associated with dominant or majority status (being White or able-bodied, for instance). These reflections brought us to use intersectionality as an interpretive lens in addition to Fraser’s theory of justice in the interpretation of findings.

### Study Design and Methodology

CBPR is defined as a research methodology that seeks to create mutually beneficial partnerships with communities that are experiencing social injustices (Wallerstein & Duran, 2010). Its purpose is to increase participation and promote equity through a process of reciprocal knowledge exchange and creation (Wallerstein & Duran, 2010). CBPR relies on fostering strong relationships built on collaboration and cooperation with the participants to ensure that they are actively involved in each step of the research process (Wallerstein, & Duran, 2010; University of British Columbia et al., 1995). In the current study, we elected to use CBPR so that adults experiencing poverty could lead the discussion on leisure activities, and regarding the necessary changes to be implemented (Wallerstein, & Duran, 2010).

### Study Setting

The organization is located in an ethnically-diverse, densely-populated urban area characterized by high material and social deprivation. Services are centred around food security, including free or low-cost breakfast and lunch provided in a large communal cafeteria serving up to 150 people, and includes psychosocial and housing-related supports through the work of outreach workers.

### Recruitment and Sampling

After the research team and the community members agreed on the use of CBPR and arts-based groups as a data collection strategy, recruitment occurred over a period of 2 weeks in June 2019. Student researchers hung advertisement posters for the in common areas at the organizations; those posters were also handed out to members by staff in the regular activities of the community organizations (e.g., food basket distribution). The selection criteria were: being at least 18 years of age, able to communicate verbally in French or English, able to sustain participation in a 2 h group-based activity and discussion, and using services at the food security organization.

### Data Collection

Participants were invited to a series of three group data collection activities, using art-based elicitation, which allowed participants to express tacit knowledge regarding their leisure experiences while engaging in arts activities (Johnson & Weller, 2002). To enhance the accessibility of the sessions for service users of the organization, a date and time was simply indicated on the advertisement poster, and the sessions were held in a common area of the organization, which was a familiar space to the service users. No prior registration was necessary, and no staff member of the community organization was present during the sessions, so as to ensure that participants felt free to comment on the services provided. When participants arrived to the session, they were presented with information and consent forms, and student researchers verbally explained the content of the forms and answered any questions. Voluntary participation was emphasized. Participants also completed a short socio-demographic questionnaire at this time.

The four student researchers led each session; two of them provided more formal instructions for the structure of the activity (one in French, one in English) while the two others participated in the activity alongside service users and were available to provide informal guidance and support. Sessions consisted of an informal artistic activity where participants could choose from a range of media (painting, drawing, letter writing, bracelet-making, other) as they were encouraged to reflect on the concept of leisure; followed by a semi-structured discussion. Discussion questions were asked in a structured yet exploratory manner (Johnson & Weller, 2002). In session 1, questions were centred around personal and experience-specific data surrounding decisions, beliefs, attitudes and emotions about leisure. In session 2, the discussions and reflections focussed on patterns of leisure engagement, and facilitators and barriers to leisure participation. In session 3, participants listed preferred past, present and future leisure activities in a chart and subsequently mapped these onto a physical map of Montreal using stickers and post-its. The three sessions lasted 2 h each.

Considering the vulnerability of this population (Shier et al., 2011) and CBPR principles, power sharing and involvement in the knowledge generation process throughout data collection was encouraged (Aldrich et al., 2017). Examples included having participants set and agree upon ground rules for sessions and accommodating arts activity preferences, other than those that had initially been planned by researchers. All sessions were audio-recorded and transcribed verbatim. Participants selected pseudonyms that were used in the data analysis and reporting. Participants received 25$ gift certificates as compensation for each session they participated in. Each student researcher also completed individual field note memos of observations during sessions, and personal journals following each session.

### Data Analysis

We conducted a thematic analysis (Smith & Firth, 2011) to describe participation and engagement in leisure. In the first stage, inductive analysis of transcripts was completed by the student researchers (Morehouse & Maykut, 2002). At minimum, each transcript was analyzed by two student researchers individually. They each interpreted transcripts by reading transcripts line-by-line, highlighting key “units of meaning” (Morehouse & Maykut, 2002). These units of meaning were triangulated with fieldnotes and reflexive journals, and coded in the margins of the transcripts using their own words (Lincoln & Guba, 1985; Smith & Firth, 2011). With subsequent re-reads, formal categories and preliminary themes emerged. In the second stage, student researchers refined these categories and preliminary themes through an iterative process of discussions with one another and their supervisor, as well as with participants from the sessions through the process of member checking. It is through this process that abstract concepts emerged, which resulted in the identification of the themes (Smith & Firth, 2011).

## Findings

### Study Participants

The sample consisted of 39 participants, including 19 men (49%), 16 women (41%) and 4 participants who reported another gender identity (10%). Participants ranged in age from 24 to 75 years old, with a mean age of 52. Nine participants identified as Middle Eastern, six as European, five participants as Canadian or Quebecer, five as Latino or Caribbean, three as White/Caucasian, two as Asians and two as Indigenous. The majority reported English (*n* = 12), French (*n* = 8) or Arabic (*n* = 7) as their first language. Although the demographic questionnaire did not collect data on health or disability status directly, 12 participants (31%) identified using physical health-related services, and nine (23%) mental health-related services.

### Themes

Six interrelated themes on the concept of leisure engagement emerged from data collected during group sessions with participants.

**“The more I do, the more alive I feel” The place of meaningful leisure activities in daily life.** Most participants discussed meaningful leisure activities that they previously engaged in, but lost as a result of immigration, physical limitations, social isolation, leisure programs discontinued by community organizations or lack of money to purchase required materials and equipment. Edith explained: “I used to play [music] but since I don’t have sheets to play, I used to play guitar and violin, if I had instruments […] it would be good not only for me but for others.”

Even though many participants experienced loss of leisure, most maintained some level of participation and purposefully chose to engage in leisure activities for various reasons. The most widely cited reason for engagement in leisure activities was that they provided opportunities for sharing and exchanging knowledge or resources. These activities helped address loneliness, connect one with the broader community and preserve social skills. Sacha, who enjoyed free concerts in the city, felt they provide a sense of being “united” with society and made her feel “love” and “joy.” Mickey suggested the potential role of community organizations as sites of leisure engagement to allow for more profound social connections:

One of the biggest problems now in the community is loneliness. […] that’s why a lot of people come here. You organize activities, why? To bring people together. So that you don’t go to this alone and that alone, you make new friends. The community organizations, you have already brought people together you just haven’t brought them *together.*

Activities that promote one’s connection with nature were valued for their wellness-enhancing qualities and as a spiritual experience. Sacha stated how it “stimulated” her “emotionally, and psychologically,” and that “the more I do […] the more alive I feel.” Finally, many participants engaged in leisure activities in order to “be of help.” Pichounette, for instance, explained how she gave back to the community organization and its members by bringing her sewing machine to the community organization once a week:

I am happy that I do my sewing activity. I am happy to have taught them something. To do something with their hands. That young one, she had a dress that had not worn for two years because the strap was broken, and we fixed it. She was proud to wear it.

**“You gotta be on watch” Preoccupation with survival needs shapes time use and leisure.** Terms such as “not having money,” “always about money,” “financial obligations” or “free stuff” dominated the participants’ discourses. Survival included financial and safety preoccupations. Lack of money to pay for leisure material, services or access to facilities was predominant, but socioeconomic status also impacted leisure in other ways. Alice, for instance, was aware of the possibility to rent city bicycles for free on certain days, but couldn’t engage in that desired activity because “*you need a credit card.*” Emile provided a compelling synthesis of the situation: “What I mean is, money, budget, it’s always about money. […] If you do only little things that cost almost nothing, you’re ok.” Lack of money also changed the meaning of some activities: Sophia contrasted her experiences of shopping for leisure before she immigrated to Canada compared to her situation at the time of the study, where shopping was no longer associated with leisure but with survival. The impact of financial constraints was so profound, that when asked what leisure activity participants would like to resume, some put it in the context of survival, either as an opportunity to make money or find work. For instance, Sam explained his preference for a co-op because “[…] you can have free coffee and have art and areas you can hang out, and you’re near people who are more likely to get you work. Like doing odd jobs if you can’t work all the time.”

Daily time use was also shaped by participants’ preoccupations with safety. Anita, a grandmother and caregiver, described spending a significant amount of time securing resources for herself and her family. Her time use was also shaped by preoccupations with perceived neighbourhood threats where she felt she always “gotta be on watch.” Many participants thus spoke of spaces perceived as “safe” as facilitators to engagement in leisure activities, since their daily environments often felt unsafe and stressful. Sam talked about preferring places that were inclusive and accepting of his leisure choices, including substance use. Concerns with survival resulted for some participants in having “too much time” and being “bored” while others were over-occupied with survival activities. Joy, for instance, described how making ends meet for ensuring her family’s survival as a single mother meant that she had “no time” for fun.

**“We need to get out” Limited but valued possibilities for escaping daily life through leisure.** Many participants spoke about being and feeling “trapped” in their current physical and mental spaces. Wanting to “get out” of their neighbourhood, the city, or their house, was a shared experience. As a result, most participants were seeking escape that could take different forms. Some like Alice, wished for “an opportunity to head out for a day” while for others, like Naima, activities like “painting and drawing” provided a psychological escape from daily life. Daniel explained how “some people have very bad apartment conditions so they’d rather be outside than in their apartment all the time.” Manuel added that “we have a problem of lack of transportation, so we’re like confined to the area. All the activities that I do is walk around sometimes.”

Outdoor activities were particularly valued for the sense of escape and freedom they provided. Some mentioned the importance of getting out for wellbeing and morale. Emile described the benefit of outdoor activities as

Much more important than activities indoors. Why? Because mentally we need to get out of the city. That’s what we need. To go out in nature and go somewhere other than here all the times. Because this is poverty. It’s what isolates us because we don’t have a choice, because we don’t have the means to go on outings. We always have this inside of us and we need nature. We need air.

The desire to “get out” was closely linked to the previous theme of survival and safety. For Alice, a trip to the park provided her with a sense of “nurturing” and protection during stressful events. Financial barriers emerged as a common denominator constraining the participants’ ability to “get out.”

### “Where to go, What to do?” Participants as Experts of Their Communities

A sub-group of participants displayed an advanced understanding of their community and acknowledged that having a wide social circle helped to discover available activities in the city. Alain recognized that when “…you have no one…it is not easy. Where to go, what to do” […] and that to “connect with everyone” is a big factor in “have{ing} information.” Those participants were able to list places that provided free and affordable leisure opportunities, including details of location, cost and transportation routes. Older and less technologically savvy participants perceived libraries as a great source of information where “there are activities announced on the walls” and the location of free activities were displayed. In contrast, a younger participant spoke extensively about subculture scenes, as a means to discover music that interested him.

Knowledge and understanding of one’s community provided participants with increased choice and control over leisure activities. That knowledge enabled participants to rely less on external resources like community organizations, and to become more active in their identification and selection of activities based on their own interests. Some participants spoke about how they used or would like to use their expertise of their community to organize activities that would mobilize others to advocate for their rights, create leisure possibilities and explore opportunities outside of their neighbourhoods. For instance, participants spoke about making a film collectively to raise awareness about the injustices and oppression they experience and to share “how we live with each other.” Those participants identified how individual factors like resourcefulness, creativity and motivation shaped their ability to engage in desired activities.

**“I just feel that it’s hopeless”: Level of vulnerability influences leisure engagement.** While some participants were experts of their community, others felt less prepared to engage in leisure independently. Participants whose poverty intersected with disability, mental illness or street homelessness reported additional challenges to their leisure engagement. Brad, who slept on the streets, often spoke about his limited social environments and having “the wrong people with me” when invited to comment on his current leisure experiences. He spoke of no current involvement in leisure other than “street drinking” with others. Similarly Alex, who had autism, stated that he had “no friends” and did not participate in leisure activities.

For these participants, hopelessness dominated, such as for Noel who wanted “more fun things but I feel that those things never come, they never happen again. And I just feel that it’s hopeless for everything.” Certain members living with disabilities managed to adapt their activities to maintain participation.

**“We don’t have the schedule!” Organizational factors shaping leisure involvement.** In addition to poverty and discrimination, participants were cognizant of the impact that structural factors within the local community sector have on engagement in leisure. Local community organizations were perceived as their primary source of leisure. Participants discussed the loss of former opportunities in some community organizations, including outings, sports and access to computers. An organizational culture characterized by decision makers staying “upstairs most of the time” and “not wanting to talk about the reasons for changes” with service users was noted to affect participants’ leisure opportunities and “motivation” to engage in available activities. For example, Nana mentioned that in some community organizations, computers were not available for recreation but for “…going to work afterwards […] not for the simple fact of having happiness.”' High staff turnover and lack of stability in activity programming were also identified as barriers to leisure engagement, as participants “don’t know when [activities] starts though! We don’t have the schedule.”

## Discussion

Previous studies on occupation and poverty called for a more in-depth analysis of the relationship between those two concepts (Leadley & Hocking, 2017). Our findings indicate that experiences of poverty can influence the meaning of leisure activities, for instance by creating a desire to escape from unsatisfying or restricted physical and social environments. While poverty itself restricted leisure engagement through material deprivation, structural factors that have been previously documented as causes of poverty (Smith-Carrier, 2017) also influence access to and experiences of time use generally, and leisure activities in particular. A notable finding in the current study was how the impact of poverty on leisure engagement was modulated by other intersecting disadvantages.

Participants provided rich descriptions of the meaning they attribute to leisure occupations. Some of those descriptions attribute meaning aligned with biomedical and psychological understandings of leisure (Capaldi et al., 2014; Chiesura, 2004; Ryan et al., 2017), where leisure emerged as a way to seek respite from difficult daily environments, either physically, through going to natural spaces like parks, or mentally, through art and meditation, and as a way to improve health and wellness. Coherent with a social-psychological understanding of leisure, participants also valued engaging in social activities to connect to family and loved ones, or experience a sense of belonging to a group. Socializing was mentioned to be a key element in order to experience, enjoy and commit to participation in leisure (Orsega-Smith et al., 2007; Suto, 2013; Toepoel, 2013). Finally, in line with a more critical understanding of leisure (Glover, 2004; Hemingway, 1999) and theories of recognition (Fraser, 1996), participants also understood leisure as activities that could increase their social capital and status, and even provide them with a collective voice that might challenge the discourses on poverty. Amidst discourses that pressured participants into normative, income-generating activities, the very thought of engaging in leisure and play thus became an act of resistance (Shaw, 2001).

Participants identified many barriers to leisure engagement, and most of them were structural and organizational rather than personal or interpersonal. Some of those factors have been previously documented: material deprivation and reliance on social assistance, which barely covers the cost of basic needs (Peter & Polgar, 2020); neoliberal discourses and practices that explicitly favour income-generating activities (Smith-Carrier, 2017); loss of support network and lack of familiarity with social and physical environments due to migration (Suto, 2013; Stodolska, 2000); and stigma associated with mental illness (Klitzing, 2004; Stewart, 2019). However, the findings of the present study add to the occupational justice literature by problematizing the notion of choice in the context of leisure engagement specifically. Overall, participants could “choose” from an extremely limited pool of leisure activities that were free or very low cost, required no or little equipment, no transportation beyond what was accessible on foot, and available in local community settings. Participants also had to carefully navigate potentially physically and socially unsafe leisure spaces. This had the effect of reducing the “vision of possibilities” (Polatajko et al., 2013, p. 382) for many, and enhanced their feelings of being “trapped” and in survival mode. This adds to a growing literature in the occupational science literature that challenges the notions of occupational choice (Hammell, 2020) and opportunities (Peters & Galvaan, 2021).

Exclusion from leisure, despite a desire to engage in such activities, resulted for many in occupational imbalance (Backman, 2004). This was observed either as being over-occupied or under-occupied. For some, boredom and having “too much time” stemmed from a lack of opportunities for meaningful engagement (Marshall et al., 2019; Farnworth, 1998). Over-occupation was an issue for individuals who were caregivers, although participants noted that it was meaningful for them to engage in caregiving activities at the expense of leisure. This was particularly the case for new immigrants, a finding that also emerged in previous studies (Suto, 2013; Stodolska, 2000). This highlights the idiosyncratic nature of leisure profiles among adults experiencing poverty, and the need to consider occupational balance in the context of individuals’ priorities and needs (Hammell, 2009). This is further reflected in Suto (2013), who cautions readers to not impose western ethnocentric definition of leisure, especially when working with populations that are culturally diverse.

The findings can also be interpreted from the lens of intersectionality and intersectional disadvantages (Collins & Bilge, 2016; Crenshaw, 1989). Although participants shared low socioeconomic status as a common devalued social category, some occupied several devalued social positions that restricted their leisure engagement even further. In the current study, participants identified disability status in particular as a social category associated with exclusion from leisure. Although age did not emerge in the participants’ discourses as a social category, we could observe – for instance, through informal conversations on health concerns or use of walking aids – that older participants tended to live with multiple co-occurring mental and physical health issues, in line with previous work on the effect of poverty and housing precariousness on premature ageing (Gallo et al., 2012; Reynolds et al., 2016). The relationship between age, disability and leisure exclusion operated through multiple processes. One of those processes, at least in the local Montreal context, is the lack of provision of reliable and free public transportation for those unable to walk long distances to reach leisure spaces due to pain and other physical health issues. Although participants talked quite elusively about “unsafe” social environments for those living with psychiatric or developmental disabilities, one can assume, from the extant literature, that ableist and sanist assumptions, and ensuing discriminatory practices such as micro-agressions, also contribute to exclusion from leisure (Klitzing, 2004; Steward, 2019). Previous studies have explored the relationships between occupational outcomes and other social markers such as race/ethnicity or gender and gender identity (Murthi & Hammell, 2020; Santos et al., 2019), and it is possible that our data collection methods did not allow us to capture the full depth of the intersecting disadvantages experienced by participants (see Study Limitations).

### Practice Implications

The findings of this study raise many potential directions to shift practices related to leisure, in order to better address the occupational rights and needs of individuals living in poverty. We focus on three implications that are particularly relevant for occupational therapy practice.

First, it is clear from the findings that occupational therapists need to shift their attention from individual skills and abilities to the conditions that impede occupational engagement and performance. This has been said before in the occupational therapy literature (Gerlach et al., 2018; Hyett et al., 2019); yet, in practice, occupational therapy interventions aimed at contextual factors rather than individual skills still appear as a second thought, or one-time projects such as student research (including the one presented here) or role-emerging placements. We argue that this needs to be reversed; as the findings from this study and others indicate that contextual factors are the primary barrier to occupational engagement, working at the level of structures, and communities should be the primary target of interventions. We wonder if, in some contexts, individual-focused occupational therapy positions should not be transformed to positions in spaces where decisions about leisure opportunities are taken (e.g., city planning). Occupational therapists should also develop the structural competencies to act in collaboration with the community sector and persons experiencing poverty. These competencies include recognizing and acting on the structures that create inequitable health and occupational outcomes, such as resource allocation, legislations or availability of services (Metzl & Hansen, 2014).

The second practice implication stems from recognizing the community expertise held by many participants. Whether expertise came from knowing others, finding out about activities online or getting involved in the subculture scene, many participants had an in-depth understanding of their community. Previous studies have documented that large social networks, and actively sought-out new opportunities increase access to knowledge and resources (Toepoel, 2013; Orsega-Smith et al., 2007). This runs contrary to existing literature which characterizes *all* individuals experiencing poverty as being vulnerable to social isolation (Eckhard, 2018; National Seniors Council, 2017; Toepoel, 2013; Stewart et al., 2009). Participants also reported a disconnect between themselves and organizational cultures and attitudes of service providers that are grounded on socially constructed priorities of societal contribution such as finding work, rather than individuals’ voiced needs for leisure activities. Service provision, both in community and institutional settings, should engage persons experiencing poverty in decision making and leisure planning. In the current CBPR study, a map of leisure activities and locations was identified as a need. The map was meant to act as a resource to enable and facilitate self-efficacy for adults experiencing poverty, ultimately helping them to better plan and locate meaningful and accessible leisure activities. Prototypes of the map were created iteratively and distributed to members of the community organization, in order to engage them in its design and ensure that the map would be functional and align with their needs and priorities. An increase in agency was observed, as the more individuals were given the opportunity to contribute their knowledge, the more engaged they became in the process.

The third implication is the need to create and sustain leisure spaces that are socially accessible. This includes spaces that are accepting of others’ occupational choices, and intersecting social identities. One participant spoke extensively about his preference for non-normative activities situated in locations considered marginal, including music venues, bars and co-ops. This is consistent with previous research that individuals in poverty seek “judgement-free spaces” (Trussell & Mair, 2010; Stewart, 2019) when looking for leisure opportunities. These are spaces where participants feel safe, connected and accepted without judgment, which may lead to increased control over activities (Trussell & Mair, 2010). These might include both dedicated (e.g., for participants sharing some social characteristics) and non-dedicated spaces. Occupational therapists can play an active role in fostering such spaces by partnering with community organizations with expertise in serving marginalized or underserved communities, leisure or recreation service providers and scholars, and city personnel or planners. One example of such a partnership that has been well documented and implemented since 2013 in Nova Scotia is the Recreation for Mental Health (R4MH) project, which aims to build inclusive recreation spaces for people with mental illness through participatory planning and integrated knowledge translation (Gallant et al., 2020; Lauckner et al., 2018).

### Research Implications

The findings highlight the need to better understand, including through implementation studies, the potential outcomes of strengthening the offer of meaningful leisure to individuals experiencing poverty. Given the financial and structural constraints faced by community organizations, such research may help empower community-based providers and supply organizations with resources to support the implementation of their vision and mission. Studies exploring the attitudes, experiences and strategies for leisure engagement from the perspective of service providers could also complement the findings of the current study. Finally, it is to be noted that the findings presented here relate to a pre-COVID-19 era. Future studies should document the transformation of occupational possibilities for groups experiencing poverty during and after the pandemic.

### Limitations

Our study included several limitations. Although the research team identified an individual experiencing poverty as a co-researcher at the beginning of the project, this person eventually became unreachable, and thus only staff in the community organization were present in the early phases of the participatory process. Additionally, time constraints to complete the study might have hindered the participation of service users in later phases (e.g., writing of this manuscript). Finally, the large sized groups may also not have allowed for more intimacy within the conversations. This may have acted as a barrier for anyone from either the Lesbian-Gay-Bisexual-Queer-Transgender-Two-Spirit (LGBQT2i) or Black, Indigenous and people of colour (BIPOC) community to speak of their intersected experiences of accessing leisure while being in poverty and having these additional identities.

## Conclusion

Our study, through the use of participatory approaches and art-based elicitation strategies, uncovered the impact of poverty on leisure occupations. Participants highly valued leisure but opportunities for leisure occupations were scarce and limited by systemic factors such as material deprivation, intersecting stigma, lack of socially accessible environments, and pervasive normative assumptions around the relative value of work, survival and leisure activities. Occupational therapists can and should work alongside members of underserved communities in both practice and research to uncover and address the systemic and local contextual barriers to engagement in leisure activities.

### Key Messages

- Systemic determinants of health and wellbeing, such as normative assumptions about the value of leisure activities, shape the occupational engagement of individuals experiencing poverty.
- Occupational therapists should develop the structural competencies to act in collaboration with the community sector and persons experiencing poverty, in order to enhance occupational possibilities for underserved groups.
- Individuals experiencing poverty are not a homogenous group, and attention to both their grounded expertise of their local community, and potential vulnerabilities, is paramount.
